# SARS-CoV-2 infection protects against rechallenge in rhesus macaques

**DOI:** 10.1126/science.abc4776

**Published:** 2020-05-20

**Authors:** Abishek Chandrashekar, Jinyan Liu, Amanda J. Martinot, Katherine McMahan, Noe B. Mercado, Lauren Peter, Lisa H. Tostanoski, Jingyou Yu, Zoltan Maliga, Michael Nekorchuk, Kathleen Busman-Sahay, Margaret Terry, Linda M. Wrijil, Sarah Ducat, David R. Martinez, Caroline Atyeo, Stephanie Fischinger, John S. Burke, Matthew D. Slein, Laurent Pessaint, Alex Van Ry, Jack Greenhouse, Tammy Taylor, Kelvin Blade, Anthony Cook, Brad Finneyfrock, Renita Brown, Elyse Teow, Jason Velasco, Roland Zahn, Frank Wegmann, Peter Abbink, Esther A. Bondzie, Gabriel Dagotto, Makda S. Gebre, Xuan He, Catherine Jacob-Dolan, Nicole Kordana, Zhenfeng Li, Michelle A. Lifton, Shant H. Mahrokhian, Lori F. Maxfield, Ramya Nityanandam, Joseph P. Nkolola, Aaron G. Schmidt, Andrew D. Miller, Ralph S. Baric, Galit Alter, Peter K. Sorger, Jacob D. Estes, Hanne Andersen, Mark G. Lewis, Dan H. Barouch

**Affiliations:** 1 Center for Virology and Vaccine Research, Beth Israel Deaconess Medical Center, Harvard Medical School, Boston, MA 02215, USA.; 2 Tufts University Cummings School of Veterinary Medicine, North Grafton, MA 01536, USA.; 3 Harvard Medical School, Boston, MA 02115, USA.; 4 Oregon Health & Sciences University, Beaverton, OR 97006, USA.; 5 University of North Carolina, Chapel Hill, NC 27599, USA.; 6 Ragon Institute of MGH, MIT, and Harvard, Cambridge, MA 02139, USA.; 7 Bioqual, Rockville, MD 20852, USA.; 8 Janssen Vaccines & Prevention BV, Leiden, Netherlands.; 9 Massachusetts Consortium on Pathogen Readiness, Boston, MA 02215, USA.; 10 Cornell University College of Veterinary Medicine, Ithaca, NY 14853, USA.

## Abstract

An understanding of protective immunity to severe acute respiratory syndrome coronavirus 2 (SARS-CoV-2) is critical for vaccine and public health strategies aimed at ending the global coronavirus disease 2019 (COVID-19) pandemic. A key unanswered question is whether infection with SARS-CoV-2 results in protective immunity against reexposure. We developed a rhesus macaque model of SARS-CoV-2 infection and observed that macaques had high viral loads in the upper and lower respiratory tract, humoral and cellular immune responses, and pathologic evidence of viral pneumonia. After the initial viral clearance, animals were rechallenged with SARS-CoV-2 and showed 5 log_10_ reductions in median viral loads in bronchoalveolar lavage and nasal mucosa compared with after the primary infection. Anamnestic immune responses after rechallenge suggested that protection was mediated by immunologic control. These data show that SARS-CoV-2 infection induced protective immunity against reexposure in nonhuman primates.

## Immunity from reinfection

One of the many open questions about severe acute respiratory syndrome coronavirus 2 (SARS-CoV-2) infection is whether an individual who has cleared the virus can be infected a second time and get sick. Chandrashekar *et al.* and Deng *et al.* generated rhesus macaque models of SARS-CoV-2 infection and tested whether natural SARS-CoV-2 infection could result in immunity to viral rechallenge. They found that animals indeed developed immune responses that protected against a second infection. Although there are differences between SARS-CoV-2 infection in macaques and in humans, these findings have key implications for public health and economic initiatives if validated in human studies.

*Science*, this issue p. 812, p. 818

## Teaser

SARS-CoV-2 infection was able to induce robust protective immunity against reexposure to virus in nonhuman primates.

The explosive spread of the coronavirus disease 2019 (COVID-19) pandemic has made the development of countermeasures an urgent global priority (*1*–*8*). However, our understanding of the immunopathogenesis of severe acute respiratory syndrome coronavirus 2 (SARS-CoV-2) is currently very limited. In particular, it is not yet known whether SARS-CoV-2 infection induces natural immunity that protects against reexposure in humans. Such information is critical for vaccine strategies, epidemiologic modeling, and public health approaches. To explore this question, we developed a rhesus macaque model of SARS-CoV-2 infection and assessed virologic, immunologic, and pathologic features of infection, as well as protective immunity against rechallenge.

## Virology and immunology of SARS-CoV-2 infection in rhesus macaques

**Fig. 1:**
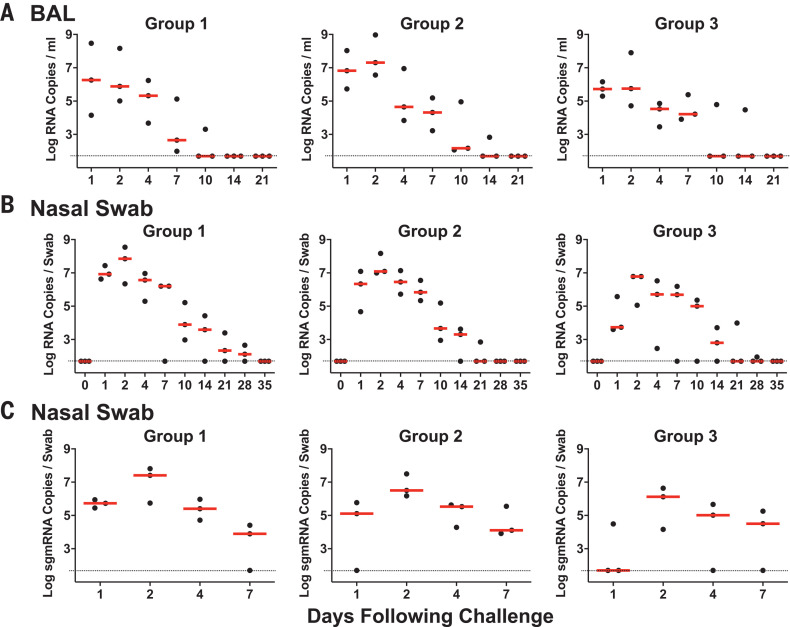
Viral loads in SARS-CoV-2–challenged rhesus macaques. Rhesus macaques were inoculated by the IN and IT routes with 1.1 × 10^6^ PFU (Group 1; *N* = 3), 1.1 × 10^5^ PFU (Group 2; *N* = 3), or 1.1 × 10^4^ PFU (Group 3; *N* = 3) of SARS-CoV-2. (**A**) Log_10_ viral RNA copies/ml (limit 50 copies/ml) were assessed in BAL at multiple time points after challenge. (**B** and **C**) Log_10_ viral RNA copies/swab (B) and log_10_ sgmRNA copies/swab (limit 50 copies/swab) (C) were assessed in NS at multiple time points after challenge. Red horizontal bars reflect median viral loads.

We inoculated nine adult rhesus macaques (6 to 12 years of age) with a total of 1.1 × 10^6^ plaque-forming units (PFU) (Group 1; *N* = 3), 1.1 × 10^5^ PFU (Group 2; *N* = 3), or 1.1 × 10^4^ PFU (Group 3; *N* = 3) of SARS-CoV-2 administered as 1 ml by the intranasal (IN) route and 1 ml by the intratracheal (IT) route. After viral challenge, we assessed viral RNA levels by reverse transcription polymerase chain reaction (RT-PCR) in multiple anatomic compartments. We observed high levels of viral RNA in bronchoalveolar lavage (BAL) (Fig. 1A) and nasal swabs (NS) (Fig. 1B), with a median peak of 6.56 (range 5.32 to 8.97) log_10_ RNA copies/ml in BAL and a median peak of 7.00 (range 5.06 to 8.55) log_10_ RNA copies/swab in NS. Viral RNA in NS increased in all animals from day 1 to day 2, suggesting viral replication. Viral RNA peaked on day 2 and typically resolved by day 10 to day 14 in BAL and by day 21 to day 28 in NS. After day 2, viral loads in BAL and NS appeared comparable in all groups regardless of dose. Viral RNA was undetectable in plasma (fig. S1). Animals exhibited modestly decreased appetite and responsiveness suggestive of mild clinical disease (fig. S2), as well as mild transient neutropenia and lymphopenia in the high-dose group (fig. S3), but fever, weight loss, respiratory distress, and mortality were not observed.

To help differentiate input challenge virus from newly replicating virus, we developed an RT-PCR assay to assess E gene subgenomic mRNA (sgmRNA), which reflects viral replication cellular intermediates that are not packaged into virions and thus represent putative replicating virus in cells (*9*). Compared with total viral RNA (Fig. 1B), sgmRNA levels were lower in NS on day 1, with a median of 5.11 (range <1.70 to 5.94) log_10_ sgmRNA copies/swab, but then increased by day 2 to a median of 6.50 (range 4.16 to 7.81) log_10_ sgmRNA copies/swab (Fig. 1C).

**Fig. 2:**
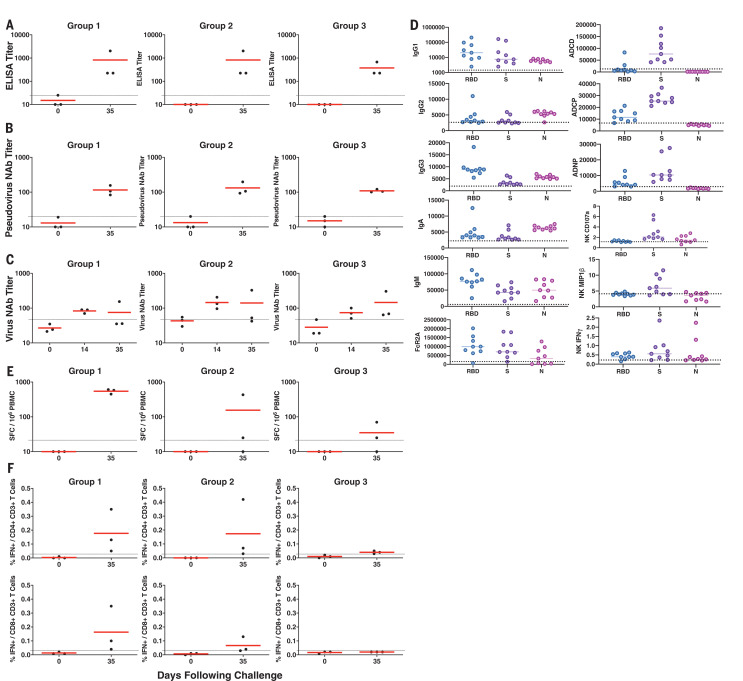
Immune responses in SARS-CoV-2–challenged rhesus macaques. (**A** to **D**) Humoral immune responses were assessed after challenge by binding antibody ELISA (A), pseudovirus neutralization assays (B), live virus neutralization assays (C), and systems serology profiles (D) including antibody subclasses and effector functions to RBD, soluble S ectodomain, and N proteins on day 35. Antibody-dependent complement deposition, antibody-dependent cellular phagocytosis, antibody-dependent neutrophil phagocytosis, and NK CD107a and cytokine secretion (NK MIP1β, NK IFNγ) are shown. (**E** and **F**) Cellular immune responses were also assessed after challenge by IFNγ ELISPOT assays (E) and multiparameter intracellular cytokine-staining assays (F) in response to pooled S peptides. Red horizontal bars reflect mean responses.

We next evaluated SARS-CoV-2–specific humoral and cellular immune responses in these animals. All nine macaques developed binding antibody responses to the SARS-CoV-2 spike (S) protein by ELISA (Fig. 2A) and neutralizing antibody (NAb) responses using both a pseudovirus neutralization assay (*10*) (Fig. 2B) and a live virus neutralization assay (*11*, *12*) (Fig. 2C). NAb titers of ~100 were observed in all animals on day 35 regardless of dose group (range 83 to 197 by the pseudovirus neutralization assay and 35 to 326 by the live virus neutralization assay). Antibody responses of multiple subclasses were observed against the receptor binding domain (RBD), the prefusion S ectodomain (S), and the nucleocapsid (N), and antibodies exhibited diverse effector functions, including antibody-dependent complement deposition, antibody-dependent cellular phagocytosis, antibody-dependent neutrophil phagocytosis, and antibody-dependent natural killer (NK) cell degranulation (NK CD107a) and cytokine secretion [NK macrophage inflammatory protein 1β (MIP1β), NK interferon γ (IFNγ)] (*13*) (Fig. 2D). Cellular immune responses to pooled S peptides were observed in most animals by IFNγ ELISPOT assays on day 35, with a trend toward lower responses in the lower-dose groups (Fig. 2E). Intracellular cytokine-staining assays demonstrated induction of both S-specific CD8^+^ and CD4^+^ T cell responses (Fig. 2F).

## SARS CoV-2 infection induces acute viral interstitial pneumonia in rhesus macaques

Only limited pathology data from SARS-CoV-2–infected humans are currently available. To assess the pathologic characteristics of SARS-CoV-2 infection in rhesus macaques, we inoculated four animals with 1.1 × 10^5^ PFU of virus by the IN and IT routes as above and necropsied them on day 2 (*N* = 2) and day 4 (*N* = 2) after challenge. Multiple regions of the upper respiratory tract, lower respiratory tract, gastrointestinal tract, lymph nodes, and other organs were harvested for virologic and pathologic analyses. High levels of viral RNA were observed in all nasal mucosa, pharynx, trachea, and lung tissues, and lower levels of virus were found in the gastrointestinal tract, liver, and kidney (fig. S4). Viral RNA was readily detected in paratracheal lymph nodes but was only sporadically found in distal lymph nodes and spleen (fig. S4).

**Fig. 3:**
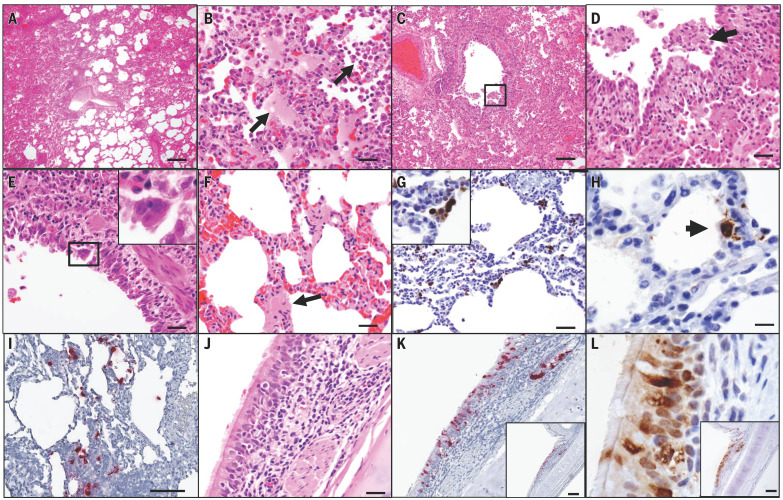
SARS-CoV-2 induces acute viral interstitial pneumonia. (**A** to **F**) Hematoxylin and eosin–stained sections of fixed lung tissue from SARS-CoV-2–infected rhesus macaques 2 days after challenge showing interstitial edema and regional lung consolidation (A), intra-alveolar edema and infiltrates of neutrophils (B), bronchiolar epithelial sloughing and necrosis [(C) and (D)], bronchiolar epithelial syncytial cell formation (E), and hyaline membranes within alveolar septa (F). (**G** and **H**) Immunohistochemistry for SARS-N showing virus-infected cells within interstitial spaces, including a viral syncytial cell within the lumen (G) and virus-infected alveolar lining cells (H). (**I**) Inflammatory infiltrate showing multiple cells containing SARS-CoV-2 RNA by RNAscope in situ hybridization. (**J** to **L**) Bronchial respiratory epithelium showing inflammation within the submucosa and transmigration of inflammatory cells into the ciliated columnar respiratory epithelium of a bronchus (J), SARS-CoV-2 RNA (K), and SARS-N (L). Scale bars: (A), 200 μm; (C), (I), (K), and (L), 100 μm; (G), 50 μm; (B), (D), (E), (F), and (J), 20 μm; (H), 10 μm.

Upper airway mucosae, trachea, and lungs were paraformaldehyde fixed, paraffin embedded, and evaluated by histopathology. On day 2 after challenge, both necropsied animals demonstrated multifocal regions of inflammation and evidence of viral pneumonia, including expansion of alveolar septae with mononuclear cell infiltrates, consolidation, and edema (Fig. 3, A and B). Regions with edema also contained numerous polymorphonuclear cells, predominantly neutrophils. Terminal bronchiolar epithelium was necrotic and sloughed with clumps of epithelial cells detected within airways and distally within alveolar spaces (Fig. 3, C and D), with formation of occasional bronchiolar epithelial syncytial cells (Fig. 3E). Hyaline membranes were occasionally observed within alveolar septa, consistent with damage to type I and type II pneumocytes (Fig. 3F). Diffusely reactive alveolar macrophages filled alveoli, and some were multinucleated and labeled positive for nucleocapsid by immunohistochemistry (Fig. 3G). Alveolar lining cells (pneumocytes) also prominently labeled positive for nucleocapsid (Fig. 3H).

Multifocal clusters of virus-infected cells were present throughout the lung parenchyma, as detected by immunohistochemistry and in situ RNA hybridization (RNAscope) (*14*, *15*) (Fig. 3I). Both positive-sense and negative-sense viral RNA were observed by RNAscope (fig. S5), suggesting viral replication in lung tissue. The dense inflammatory infiltrates included polymorphonuclear cells detected by endogenous myeloperoxidase staining, CD68^+^ and CD163^+^ macrophages, CD4^+^ and CD8^+^ T lymphocytes, and diffuse up-regulation of the type 1 IFN gene MX1 (fig. S6). SARS-CoV-2 infection led to a significant increase in polymorphonuclear cell infiltration of lung alveoli compared with uninfected animals (*P* = 0.0286), as well as extensive MX1 staining in ~30% of total lung tissue (*P* = 0.0286) (fig. S7). Inflammatory infiltrates were also detected in the respiratory epithelial submucosa of larger airways, with transmigration of inflammatory cells into bronchiole lumen (Fig. 3J). Ciliated epithelial cells also stained positive for both SARS-CoV-2 RNA (Fig. 3K) and SARS nucleocapsid (SARS-N) (Fig. 3L). By day 4 after infection, the extent of inflammation and viral pneumonia had diminished, but virus was still detected in lung parenchyma, and neutrophil infiltration and type 1 IFN responses persisted (fig. S7).

**Fig. 4:**
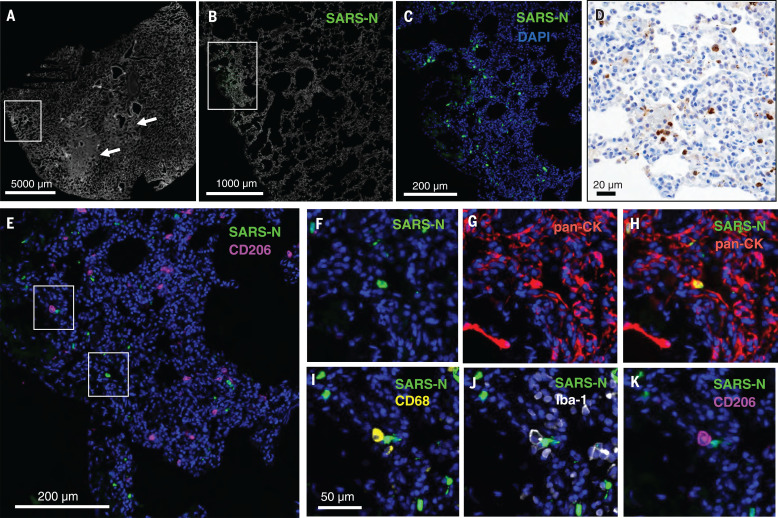
SARS-CoV-2 infects alveolar epithelial cells in rhesus macaques. Shown is CyCIF staining of fixed lung tissue from SARS-CoV-2–infected rhesus macaques 2 days after challenge. (**A**) Whole-slide image of a lung stained with Hoechst 33342 to visualize cell nuclei (grayscale); regions of nuclear consolidation (arrows) and foci of viral replication (box) are highlighted. (**B**) Higher-magnification image of inset box in (A) showing staining for SARS-N (green) and cell nuclei (grayscale). (**C**) Higher-magnification image of inset box in (B) showing SARS-N (green) and cell nuclei (blue). (**D**) Bright-field immunohistochemistry for SARS-N from corresponding lung region depicted in (C). (**E** to **K**) CyCIF staining for DNA (all panels, blue) and SARS-N [(E), (F), and (H) to (K), green], CD206 [(E) and (K), magenta], pan-CK [(G) and (H), red], CD68 [(I), yellow], or Iba-1 [(J), grayscale] showing virus-infected epithelial cells and macrophages near an infected epithelial cell. Scale bar for (F) to (K), 50 μm.

To further characterize infected tissues, we performed cyclic immunofluorescence (CyCIF) imaging, a method for multiplex immunophenotyping of paraformaldehyde-fixed tissue specimens (*16*). Tissues were stained for SARS-N, pan-cytokeratin (to identify epithelial cells), Iba-1 (ionized calcium-binding adaptor as a pan-macrophage marker), CD68 (monocyte and macrophage marker), and CD206 (macrophage marker), in addition to a panel of markers to identify other immune cells and anatomical structures (table S1) and counterstaining for DNA to label all nuclei. Foci of virus-infected cells were randomly dispersed throughout the lung and were variably associated with inflammatory infiltrates (Fig. 4, A to D). Some areas of parenchymal consolidation and inflammation contained little to no virus (Fig. 4A, arrows, and fig. S8). Virus-infected cells frequently costained with pan-cytokeratin (Fig. 4, E to H), suggesting that they were alveolar epithelial cells (pneumocytes). Uninfected Iba-1^+^ CD68^+^ CD206^+^ activated macrophages were also frequently detected adjacent to virally infected epithelial cells (Fig. 4, E and I to K). These data demonstrate that SARS-CoV-2 induced multifocal areas of acute inflammation and viral pneumonia involving infected pneumocytes, ciliated bronchial epithelial cells, and likely other cell types.

## Protective efficacy against rechallenge with SARS-CoV-2 in rhesus macaques

**Fig. 5:**
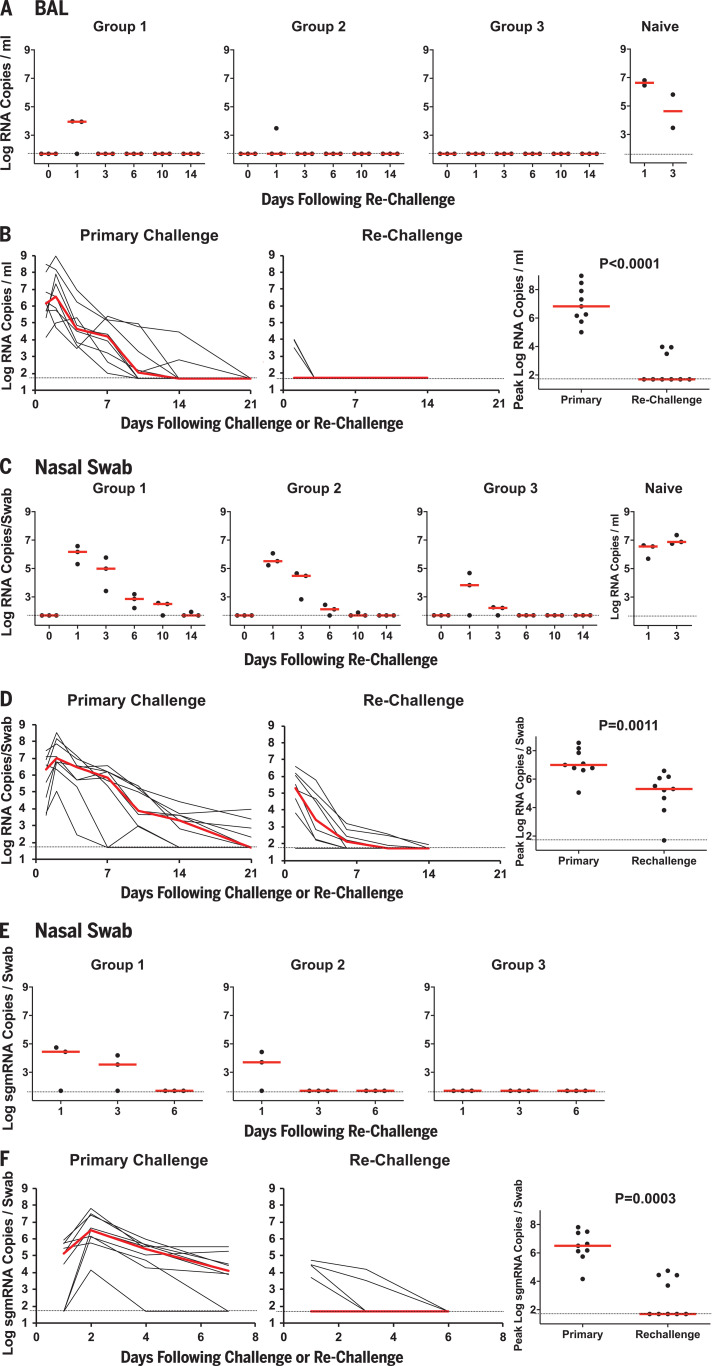
Viral loads after SARS-CoV-2 rechallenge in rhesus macaques. On day 35 after the initial infection (Fig. 1), rhesus macaques were rechallenged by the IN and IT routes with 1.1 × 10^6^ PFU (Group 1; *N* = 3), 1.1 × 10^5^ PFU (Group 2; *N* = 3), or 1.1 × 10^4^ PFU (Group 3; *N* = 3) of SARS-CoV-2. Three naïve animals were included as a positive control in the rechallenge experiment. (**A**) Log_10_ viral RNA copies/ml (limit 50 copies/ml) were assessed in BAL at multiple time points after rechallenge. One of the naïve animals could not be lavaged. (**B**) Comparison of viral RNA in BAL after primary challenge and rechallenge. (**C** and **E**) Log_10_ viral RNA copies/ml (C) and log_10_ sgmRNA copies/swab (limit 50 copies/ml) (E) were assessed in NS at multiple time points after rechallenge. (**D** and **F**) Comparison of viral RNA (D) and sgmRNA (F) in NS after primary challenge and rechallenge. Red horizontal bars reflect median viral loads. *P* values reflect two-sided Mann-Whitney tests.

On day 35 after initial viral infection (Figs. 1 and 2), all nine rhesus macaques were rechallenged with the same doses of SARS-CoV-2 that were used for the primary infection, namely 1.1 × 10^6^ PFU (Group 1; *N* = 3), 1.1 × 10^5^ PFU (Group 2; *N* = 3), or 1.1 × 10^4^ PFU (Group 3; *N* = 3). Three naïve animals were included as positive controls in the rechallenge experiment. Very limited viral RNA was observed in BAL on day 1 after rechallenge in two Group 1 animals and in one Group 2 animal, with no viral RNA detected at subsequent time points (Fig. 5A). By contrast, high levels of viral RNA were observed in the concurrently challenged naïve animals (Fig. 5A), as expected. Median peak viral loads in BAL were >5.1 log_10_ lower after rechallenge compared with after the primary challenge (*P* < 0.0001, two-sided Mann-Whitney test; Fig. 5B). After rechallenge, viral RNA was higher in NS compared with BAL but exhibited dose dependence and rapid decline (Fig. 5C), and median peak viral loads in NS were still >1.7 log_10_ compared with after the primary challenge (*P* = 0.0011, two-sided Mann-Whitney test; Fig. 5D).

We speculated that most of the virus detected in NS after rechallenge was input challenge virus, so sgmRNA levels in NS were assessed. Low but detectable levels of sgmRNA were still observed in four of nine animals in NS on day 1 after rechallenge, but sgmRNA levels declined quickly (Fig. 5E) and median peak sgmRNA levels in NS were >4.8 log_10_ lower after rechallenge compared with after the primary challenge (*P* = 0.0003, two-sided Mann-Whitney test; Fig. 5F). Consistent with these data, plaque assays in BAL and NS samples after rechallenge showed no recoverable virus and plaque levels were lower than those after the primary infection (*P* = 0.009 and *P* = 0.002, respectively, two-sided Mann-Whitney tests; fig. S9). Moreover, little or no clinical disease was observed in the animals after rechallenge (fig. S10).

**Fig. 6:**
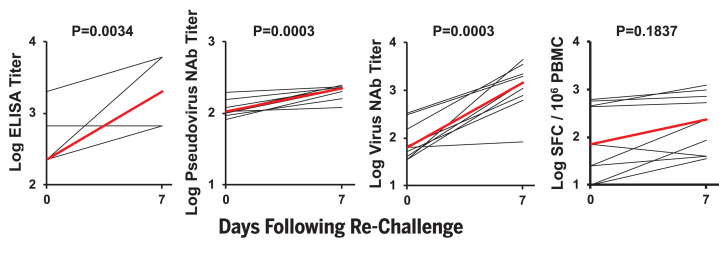
Anamnestic immune responses after SARS-CoV-2 rechallenge in rhesus macaques. Results of binding antibody ELISAs, pseudovirus neutralization assays, live virus neutralization assays, and IFNγ ELISPOT assays are depicted before and 7 days after SARS-CoV-2 rechallenge. Red lines reflect mean responses. *P* values reflect two-sided Mann-Whitney tests.

After SARS-CoV-2 rechallenge, animals exhibited rapid anamnestic immune responses, including increased virus-specific ELISA titers (*P* = 0.0034, two-sided Mann-Whitney test), pseudovirus NAb titers (*P* = 0.0003), and live virus NAb titers (*P* = 0.0003), as well as a trend toward increased IFN-γ ELISPOT responses (*P* = 0.1837) by day 7 after rechallenge (Fig. 6). In particular, NAb titers were markedly higher on day 14 after rechallenge compared with day 14 after the primary challenge (*P* < 0.0001, two-sided Mann-Whitney test) (fig. S11). All animals developed anamnestic antibody responses after rechallenge regardless of the presence or absence of residual viral RNA or sgmRNA in BAL or NS, so we speculate that the protective efficacy against rechallenge was mediated by rapid immunologic control.

## Discussion

Individuals who recover from certain viral infections typically develop virus-specific antibody responses that provide robust protective immunity against reexposure, but some viruses, such as HIV-1 (*17*), do not generate protective natural immunity. Human challenge studies for the common cold coronavirus 229E have suggested that there may be partial natural immunity (*18*). However, there are currently no data on whether humans who have recovered from SARS-CoV-2 infection are protected from reexposure (*19*). This is a critical issue with profound implications for vaccine development, public health strategies, antibody-based therapeutics, and epidemiologic modeling of herd immunity. In this study, we have demonstrated that SARS-CoV-2 infection in rhesus macaques provides protective efficacy against SARS-CoV-2 rechallenge.

We developed a rhesus macaque model of SARS-CoV-2 infection that recapitulates many aspects of human SARS-CoV-2 infection, including high levels of viral replication in the upper and lower respiratory tract (Fig. 1) and clear pathologic evidence of viral pneumonia (Figs. 3 and 4). Histopathology, immunohistochemistry, RNAscope, and CyCIF imaging demonstrated multifocal clusters of virus-infected cells in areas of acute inflammation, with evidence for virus infection of alveolar pneumocytes and ciliated bronchial epithelial cells. These data suggest the utility of rhesus macaques as a model for testing vaccines and therapeutics and for studying the immunopathogenesis of SARS-CoV-2 infection, and our findings complement and extend recently published data in cynomolgus macaques (*20*). However, neither nonhuman primate model led to respiratory failure or mortality, so further research will be required to develop a model of severe COVID-19 disease.

SARS-CoV-2 infection in rhesus macaques led to humoral and cellular immune responses (Fig. 2) and provided protection against rechallenge (Fig. 5). Residual low levels of subgenomic mRNA in nasal swabs in a subset of animals (Fig. 5) and anamnestic immune responses in all animals (Fig. 6) after SARS-CoV-2 rechallenge suggest that protection was mediated by immunologic control and likely was not sterilizing.

Given the near-complete protection in all animals after SARS-CoV-2 rechallenge, we were unable to determine immune correlates of protection in this study. SARS-CoV-2 infection in rhesus monkeys resulted in the induction of neutralizing antibody titers of ~100 as measured by both a pseudovirus neutralization assay and a live virus neutralization assay, but the relative importance of neutralizing antibodies, other functional antibodies, cellular immunity, and innate immunity to protective efficacy against SARS-CoV-2 remains to be determined. Moreover, additional research will be required to define the durability of natural immunity.

In summary, SARS-CoV-2 infection in rhesus macaques induced humoral and cellular immune responses and provided protective efficacy against SARS-CoV-2 rechallenge. These data raise the possibility that immunologic approaches to the prevention and treatment of SARS-CoV-2 infection may in fact be possible. However, it is critical to emphasize that there are important differences between SARS-CoV-2 infection in macaques and humans, with many parameters still yet to be defined in both species, so our data should be interpreted cautiously. Rigorous clinical studies will be required to determine whether SARS-CoV-2 infection effectively protects against SARS-CoV-2 reexposure in humans.

## Supplementary Materials

science.sciencemag.org/content/369/6505/812/suppl/DC1 Materials and Methods Table S1 Figs. S1 to S11 References

## Appendix

View/request a protocol for this paper from *Bio-protocol*.
